# Association of General Obesity and Abdominal Obesity with the Prevalence of Urinary Incontinence in Women: Cross-sectional Secondary Data Analysis

**Published:** 2018-06

**Authors:** Sunah PARK, Kyoung Ah BAEK

**Affiliations:** 1. Dept. of Nursing, Gangneung-Wonju National University, Wonju, Republic of Korea; 2. Dept. of Nursing, Kookje University, Gyeonggi-do, Republic of Korea

**Keywords:** Obesity, Urinary incontinence, Women

## Abstract

**Background:**

Urinary incontinence is prevalent among older adults worldwide and associated with lower quality of life. Obesity is highly associated with development or exacerbation of urinary incontinence. We examined the impact of different types of obesity (general obesity and abdominal obesity) on urinary incontinence.

**Methods:**

We employed 2007–2009 Korean National Health and Nutrition Examination Survey (KNHANES) with 4648 females over 19 yr of age. Body mass index, waist circumstance, total body fat percentage, trunk fat percentage, demographic variables, and potential confounding factors were assessed. Chi-square tests and logistic regression analysis were used.

**Results:**

There were significant trends of increasing risk of urinary incontinence with increasing body mass index (*P* =.002), waist circumstance (*P* = .001), percent total body fat (*P* =.029) and percent trunk fat (*P* =.005). Regarding the association of urinary incontinence prevalence with different types of obesity, nonobese women with abdominal obesity had the highest odds ratio of urinary incontinence, followed by obese women with abdominal obesity (odds ratio = 1.59 and 1.55, respectively).

**Conclusion:**

Abdominal obesity may be more likely to be associated with urinary incontinence compared to overall obesity. Early screening and identification of abdominal obesity may be needed for older women to prevent or reduce urinary incontinence episodes.

## Introduction

Urinary incontinence (UI), defined as any involuntary leakage of urine, is prevalent among older adults worldwide (1). The Fourth International Consultation on Incontinence reported that the worldwide prevalence of UI ranged from 25% to 45% in women (1). In Korea, the prevalence of UI ranges from 45% to 64.6% and increased with age, up to 70.5% (2, 3). UI can lead to psychological distress such as shame, anxiety, and depression (4) as well as severe restrictions to an active social life and a lower quality of life (5).

Obesity, which is becoming a worldwide health problem, is a risk factor for many chronic diseases (6). In particular, obesity is highly associated with development or exacerbation of UI with increasing intra-abdominal pressure or weakening of the pelvic floor muscle (7).

Most studies identified the association between UI and obesity by measuring body mass index (BMI), waist circumference (WC), or waist to hip ratio (8–11). BMI generally represents general obesity, whereas WC and waist to hip ratio are measures of abdominal obesity. BMI has been established as a risk factor for UI over the past decades (12). However, increased WC, rather than BMI, has been shown to be associated with the prevalence of UI when both BMI and WC are included in the same analysis model (9, 13, 14). The effects of obesity on UI may be variable. BMI is widely used as a surrogate measurement of obesity, but it is uncertain whether BMI represents an accurate body-fat distribution (10). For example, people with normal BMI might have a higher percentage of body fat, a condition known as normal-weight obesity (15). BMI also cannot differentiate between muscle and fat (15). WC has been used to determine the distribution pattern of body fat, but it is also an indirect method of determining fat distribution (16). Little information is available about the associations between UI and body-fat distribution using an accurate measurement.

Therefore, to investigate the association between UI and obesity, this study examined the impact of different types of obesity (general obesity and abdominal obesity) on UI. Additionally, this study investigated the relationship between UI and fat distribution measured by dual-energy x-ray absorptiometry (DXA), which can provide a direct determination of total body fat mass and trunk fat mass.

## Methods

### Data Sources and Sampling

We used data from the Korean National Health and Nutrition Examination Survey (KNHANES), which is a cross-sectional, nationwide, population-based survey. The KNHANES has been conducted in 1998, 2001, 2005, 2007–2009, 2010–2012, and 2013–2015. Through all of the KNHANES, a questionnaire related to UI was employed only in the KNHANES IV; thus, this study used the data from the KNHANES IV (2007–2009).

The KNHANES was established by the Korea Centers for Disease Control and designed to assess and evaluate the overall health and nutritional status of representative Koreans; it consists of 3 parts: a health-related survey, a nutrition-related survey, and a physical examination (17). The health interview and health examination are conducted by trained staff members, including physicians, medical technicians, and health interviewers (17). The surveys include detailed information on socioeconomic status, health behaviors, quality of life, healthcare utilization, biochemical profiles using fasting blood serum and urine, radiography results, food intake and dietary behavior, and measures of dental health, vision, hearing, and bone density (17). A multistage clustered probability sampling of noninstitutionalized household members was used for a representative sample. Each survey year includes a new sample of approximately 10000 individuals aged 1 year and over (17).

In the KNHANES IV (2007–2009), approximately 24871 individuals (78.4%) participated in at least 1 of the 3 parts of the data collection (i.e., health interview, physical examination, and nutrition survey). In this study, the sample was limited to women aged 19 and older without chronic renal failure. Of 24871 subjects, 8548 satisfied these inclusion criteria. The total number of valid cases was 4648 after excluding those with missing data (n= 3900), including no answers or failure of a physical examination (e.g., weight, height, WC, and whole-body percent fat).

### Measurements

UI status was defined according to the following question in the 2007–2009 KNHANES questionnaires: “Do you have urinary incontinence?” Possible responses were yes, no, or not applicable. Obesity was defined using BMI and WC as well as total body fat and trunk fat percentage. BMI was calculated as weight (kg) divided by the square of height (m). WC was measured to the nearest 0.1 cm in a horizontal plane at the level of the midpoint between the iliac crest and the costal margin at the end of expiration. Total body fat and trunk fat were estimated by DXA (Discovery-W; Hologic Inc., MA, USA) to determine body fat distribution. Percent body fat for the whole body and trunk region was calculated as fat mass divided by total mass times 100. General obesity was defined as a BMI of ≥25 kg/m (2, 18) and abdominal obesity was defined as a WC ≥80 cm (19).

Demographic variables included marital status, education level, income, residential area, and job. Confounding variables of UI and obesity included smoking, drinking, exercise, parity, diabetes, blood pressure (BP), metabolic syndrome, energy intake, and fat intake. Smoking and drinking were specifically surveyed through self-report questionnaires. A current smoker was defined as a subject who was smoking at the time of survey. A heavy drinker was defined as a subject who reported drinking more than 3 bottles each week based on a daily consumption of 30 gr of alcohol. Regular exercise was defined as exercise more than 3 times per week for greater than 30 min each time. Total energy and fat intake was computed from total energy and fat intake found in the survey by visiting the subject at home to perform a recall of food intake for the past 24 h. Hypertension (HTN) was defined as a systolic BP ≥140 mmHg and/or a diastolic BP ≥90 mmHg or the use of antihypertensive medication for BP control. Diabetes mellitus (DM) was defined as fasting blood glucose ≥126 mg/dL or being under treatment for the disease. Metabolic syndrome was defined based on the criteria proposed by the American Heart Association; the National Heart, Lung, and Blood Institute; and the International Diabetes Federation (20). Metabolic syndrome was diagnosed when 3 or more of the following criteria were met: 1) WC ≥80 cm in women; 2) systolic BP and/or diastolic BP values ≥130/85 mmHg or being on antihypertensive drug treatment and with a history of hypertension; 3) fasting glucose ≥100 mg/dL or being on drug treatment for elevated glucose; 4) fasting serum triglycerides ≥150 mg/dL or being on a relevant medication; and 5) high-density lipoprotein cholesterol <50 mg/dL in women or being on relevant medication.

### Data Analysis

All data are presented as means and SE or as proportions. Descriptive analysis was used to assess the characteristics of subjects.

To examine the differences in BMI, WC, total body fat, and trunk fat with the prevalence of UI, χ^2^ analysis was employed. Multivariate logistic regression analyses were used to estimate the odds ratios (ORs) and 95% confidence intervals (CIs) to examine the associations between the prevalence of UI and obesity. Statistical analyses were performed using the SAS survey procedure (version 9.2; SAS Institute, Inc., Cary, NC, USA) to reflect the complex sampling design. Statistical significance was considered for *P* values was <.05.

## Results

### Characteristics of Participants

The characteristics of the participants are summarized in Table 1. Most women with UI were aged 40 yr or older (86.0%, *P* < .001). Women with UI had a significantly (*P*= .002) higher parity than women without UI. Nearly half of incontinent women (47.0%) had a lower education level than a high school diploma. No significant differences in residential area, income, marital status, occupation, or smoking status were found between continent and incontinent women. Women with UI had higher prevalence’s of DM (*P*= .021), HTN (*P*= .010), and metabolic syndrome (*P*<.001). There were significant differences between continent and incontinent women in BMI (*P*<.001), WC (*P*< .001), and trunk fat percentage (*P*= .001). However, the total body fat percentage was not significantly different between continent and incontinent women.

**Table 1:** Characteristics of participants

***Characteristics***	***Urinary incontinence^a^***		***P-value^b^***
	**No (n = 4227)**	**Yes (n = 421)**	
Age (yr)			
19–39	28.1 (1.1)	13.9 (2.0)	<.001
40–64	53.7 (1.1)	66.0 (2.8)	
≥65	18.1 (0.8)	20.0 (2.4)	
Parity	3.8 ± 0.0	4.2 ± 0.1	.002
Regular exercise^c^	25.1 (1.0)	31.1 (2.4)	.012
Residential area (urban)	79.3 (2.1)	78.3 (2.8)	.692
Education (at least high school)	56.7 (1.1)	47.0 (3.1)	.001
Income (lowest quartile)	18.3 (0.9)	19.8 (2.2)	.493
Marital status (married)	79.7 (0.9)	82.3 (2.1)	.263
Occupation	44.2 (1.0)	49.7 (2.7)	.051
Current smoker	5.3 (0.4)	5.1 (1.3)	.870
Heavy drinker^d^	4.8 (0.4)	2.0 (0.8)	.020
DM	8.2 (0.5)	12.1 (1.9)	.021
HTN	26.4 (0.9)	33.4 (2.8)	.010
Metabolic syndrome	27.2 (1.0)	37.1 (2.6)	<.001
Energy intake (kcal)	1606.6 ± 12.8	1650.4 ± 31.7	.201
Fat intake (%)	15.4 ± 0.2	14.3 ± 0.4	.006
BMI (kg/m^2^)	23.4 ± 0.1	24.1 ± 0.2	<.001
WC (cm)	78.8 ± 0.2	81.8 ± 0.5	<.001
Fat (%)^e^	32.5 ± 0.1	33.1 ± 0.3	.065
Trunk fat (%)^e^	32.8 ± 0.2	34.1 ± 0.4	.001

Abbreviations: DM, diabetes mellitus; HTN, hypertension; BMI, body mass index; WC, waist circumference.

a Results are shown as percent (SE) or mean ± SE //

b *P*-values obtained by χ^2^ test or *t*-test

c Regular exercise: >30 min at a time, more than 3 times per week

d Heavy drinker: >30 g alcohol per day //

e Measured by dual-energy x-ray absorptiometry

### Prevalence of UI according to BMI, WC, percent total body fat, and percent trunk fat

Table 2 showed the prevalence of UI according to BMI, WC, percent total body fat, and percent trunk body fat. The prevalence of UI was significantly increased when BMI and WC increased (*P* < .001 for each). Similarly, the prevalence of UI exhibited an increasing trend in relation to percent total body fat and percent trunk fat (*P* = .039 and *P* = .008, respectively).

**Table 2:** Differences between prevalence of urinary incontinence and obesity-related factors

***Variable***	***Urinary incontinence***	
	**% (SE)**	***P*-value a**
BMI (kg/m^2^)		
<18.5	3.2 (1.5)	<.001
≥18.5–<23	7.0 (0.6)	
≥23–<25	9.6 (1.0)	
≥25	10.6 (1.0)	
WC (cm)		
<80	6.6 (0.5)	<.001
≥80	10.8 (0.8)	
Total body fat (%)		
T1 (<30.5)	6.8 (0.7)	.039
T2 (≥30.5–<34.9)	9.3 (0.8)	
T3 (≥34.9)	9.4 (1.0)	
Trunk fat (%)		
T1 (<30.4)	6.6 (0.7)	.008
T2 (≥30.4–<36.6)	8.5 (0.8)	
T3 (≥36.6)	10.2 (1.0)	

Abbreviations: BMI, body mass index; T, tertile; WC, waist circumference //

a *P*-values obtained by χ^2^ test.

### Associations between BMI, WC, total body fat and trunk fat, and UI

We used logistic regression analysis to determine whether UI can be predicted based on BMI, WC, total body fat, and trunk fat (Table 2). Women in the highest tertile of trunk fat were at the greatest risk for UI. In model 1, after adjusting for age, high BMI (≥25 kg/m^2^), high WC (≥80 cm), and the highest tertile of trunk fat (T3) were significantly associated with UI (OR, 95% CI: 1.42, 1.08–1.87; 1.52, 1.21–1.91; and 1.53, 1.13–2.08, respectively). These associations were still present after adjusting for all potential confounding factors (model 3).

**Fig. 1:**
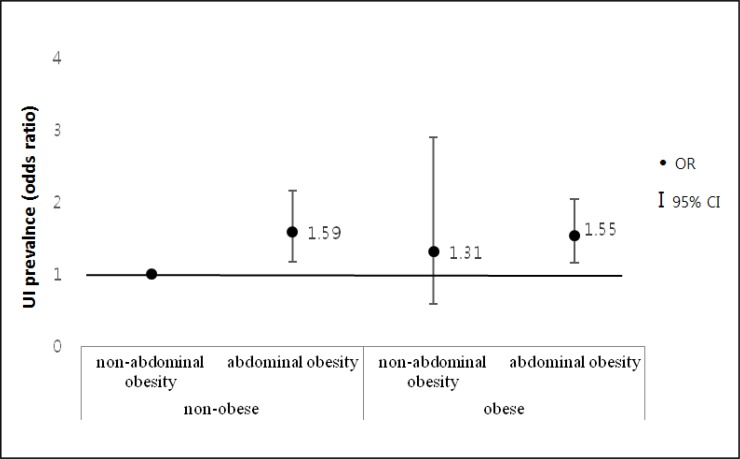
Prevalence of UI in 4 types of overall and abdominal obesity Nonobese was defined as body mass index (BMI) <25 kg/m^2^ and obese as BMI ≥25 kg/m^2^. Non-abdominal obesity was defined as waist circumference (WC) <80 cm and abdominal obesity as WC ≥80 cm

We also analyzed the association between UI prevalence and 4 types of obesity by estimating adjusted ORs in model 3 (Fig. 1) (Table 3).

**Table 3:** Adjusted ORs of the prevalence of UI according to obesity-related factors^a^

	***Urinary incontinence, OR (95% CI)***		
	**Model 1**	**Model 2**	**Model 3**
BMI (kg/m^2^)			
<18.5	0.47 (0.18–1.24)	0.47 (0.17–1.30)	0.46 (0.17–1.28)
≥18.5–<23	1.00	1.00	1.00
≥23–<25	1.37 (1.01–1.85)	1.35 (1.00–1.84)	1.35 (0.99–1.84)
≥25	1.42 (1.08–1.87)	1.44 (1.08–1.92)	1.43 (1.07–1.93)
*P* value for trend	.001	.001	.002
WC (cm)			
<80	1.00	1.00	1.00
≥80	1.52 (1.21–1.91)	1.55 (1.22–1.97)	1.54 (1.21–1.97)
*P* value for trend	<.001	<.001	.001
Total body fat (%)			
T1 (<30.5)	1.00	1.00	1.00
T2 (≥30.5–<34.9)	1.39 (1.05–1.85)	1.40 (1.06–1.85)	1.45 (1.10–1.93)
T3 (≥34.9)	1.38 (0.99–1.92)	1.41 (1.01–1.97)	1.48 (1.05–2.09)
*P* value for trend	.058	.046	.029
Trunk fat (%)			
T1 (<30.4)	1.00	1.00	1.00
T2 (≥30.4–<36.6)	1.25 (0.92–1.71)	1.28 (0.95–1.74)	1.26 (0.93–1.71)
T3 (≥36.6)	1.53 (1.13–2.08)	1.57 (1.15–2.13)	1.60 (1.16–2.20)
*P* value for trend	.007	.004	.005

Abbreviations: BMI, body mass index; CI, confidence interval; OR, odds ratio; T, tertile; WC, waist circumference.

a Model 1 was adjusted for age; model 2 was adjusted as for model 1 plus smoking, drinking, exercise, income, and education; and model 3 was adjusted as for model 2 plus parity, energy intake, and fat intake.

We divided the subjects into the following 4 groups: non-obese without abdominal obesity, non-obese with abdominal obesity, obese without abdominal obesity, and obese with abdominal obesity. Nonobese women with abdominal obesity had the highest OR for UI, followed by obese women with abdominal obesity (ORs, 1.59 and OR = 1.55, respectively).

## Discussion

By using a nationwide sample from the Korean population, we examined the associations between UI and different types of obesity. We also investigated the association between UI and total body fat and trunk fat percentages measured using DXA.

Our findings confirmed that general obesity (BMI) and abdominal obesity (WC) are associated with the prevalence of UI, consistent with previous studies (8–14, 21). However, evaluation of the association between UI and the 4 types of obesity showed that women with a high WC (≥80 cm) and normal BMI were significantly more likely to report UI symptoms than obese women (BMI ≥25 kg/m^2^) with a high or low WC, suggesting that abdominal obesity might have a greater impact on UI than general obesity. Similarly, a Brazilian study of 1069 women ≥60 yr of age found that WC, but not BMI, is associated with a higher prevalence of UI (14). WC and the waist to hip ratio were related to UI even after adjusting for BMI (10, 22). In addition, the severity of UI is known to be related to central rather than peripheral obesity (13, 23). Excess fat accumulation in the abdominal area is more likely to directly increase pressure on the pelvis (24) than high BMI, which then leads to UI.

General obesity as measured by BMI is strongly associated with the incidence of any type of UI (8, 25). However, previous studies on this subject are limited by the fact that they did not control for WC in their analyses. In addition, BMI does not reflect changes in body fat distribution, such as abdominal obesity, although an increase in BMI could attribute to increased WC. In fact, obesity types measured by BMI vs. WC have different impacts on UI (10). Specifically, increased BMI might be linked to mobility limitations or diabetes with increased urine production and urinary frequency, resulting in mixed and urge UI, while abdominal adiposity increases intra-abdominal pressure, which contributes to stress UI (10, 26). Further research using longitudinal UI data in addition to data regarding UI subtypes is needed to better understand how different types of obesity affect the occurrence of UI.

To our knowledge, this is first report of associations between UI and body fat percentage used as a measure of body fat distribution. We found the same trends as those reported for BMI and WC in multivariate logistic regression analyses. BMI and WC might be surrogate measurements used to estimate body fat mass. In addition, measuring body fat percentage might be considered as an alternative approach used to assess obesity.

Our findings suggest that weight loss, especially a reduction in abdominal fat, might be important for preventing or reducing UI. Weight reduction is reported to be associated with a significant decrease in UI symptoms (27, 28). In a large randomized trial involving 338 overweight and obese women (an intensive 6-month behavioral weight-loss program vs a structured education program), the mean number of weekly UI episodes decreased by 47% in the intervention group, but only by 28% in the controls (27). Another prospective longitudinal study reported that a weight reduction of 5%–10% is associated with significant improvements in a pad test (28). Further research is required to examine the effects of weight reduction, and specifically decreases in abdominal obesity on UI symptoms.

Our study had some potential limitations. First, UI might have been underreported because the data were self-reported, although the interview was conducted by trained health interviewers. Participants responding to UI surveys might have a tendency to keep their condition as private as possible due to embarrassment. Therefore, examiners should be trained to use more carefully worded questionnaires. Second, we are unable to address the causal relationship between obesity and the prevalence of UI because of the cross-sectional design of the study. A longitudinal study may be required to determine how obesity affects the development of UI.

## Conclusion

Obesity, especially abdominal obesity, may be an important risk factor for UI. Weight loss would be an effective approach for improving UI symptoms in obese women with UI. Early screening and identification of abdominal obesity may also be needed for older women to prevent UI episodes.

## Ethical considerations

Ethical issues (Including plagiarism, informed consent, misconduct, data fabrication and/or falsification, double publication and/or submission, redundancy, etc.) have been completely observed by the authors.
